# The Impact of Fluid Resuscitation on Clinical Outcomes According to Transport Time in Out-of-Hospital Cardiac Arrest Patients

**DOI:** 10.3390/jcm14092867

**Published:** 2025-04-22

**Authors:** Eujene Jung, Young Sun Ro, Kyoung Jun Song, Sang Do Shin, Hyun Ho Ryu

**Affiliations:** 1 Department of Emergency Medicine, Chonnam National University Medical School, Jeollanamdo 58128, Republic of Korea; em.jung.eujene@gmail.com; 2 Department of Emergency Medicine, Seoul National University, Seoul 03080, Republic of Korea; em.ro.youngsun@gmail.com (Y.S.R.); sdshin@snu.ac.kr (S.D.S.); 3 Department of Emergency Medicine, Seoul National University Boramae Hospital, Seongnam 13620, Republic of Korea; skciva@gmail.com

**Keywords:** fluid, heart arrest, cardiac arrest, transport, outcome

## Abstract

**Background/Objectives**: Current guidelines recommend fluid resuscitation only for out-of-hospital cardiac arrest (OHCA) patients with hypovolemia. This study aimed to determine whether intravenous (IV) fluid resuscitation improves survival outcomes of OHCA patients and how fluid resuscitation is influenced by the emergency medical service (EMS)-treated time interval (ETI), including scene time interval and transport time interval. **Methods**: EMS-treated OHCA adult patients with presumed cardiac etiology were enrolled between 2018 and 2019. The main exposure was IV fluid resuscitation by an EMS provider during transportation. The main outcomes were survival to discharge and neurological recovery. Multivariable logistic regression analysis calculated adjusted odd ratios (aORs). Interaction analysis between IV fluid resuscitation and ETI was also performed. **Results**: Of 29,228 eligible patients, 13,683 (46.8%) patients received IV fluid resuscitation. Patients receiving IV fluid resuscitation had a significantly higher likelihood of survival to discharge (aOR [95% confidence interval, CI]: 1.15 [1.01–1.32]). Considering the interaction effects between IV fluid resuscitation and ETI for survival to discharge, aOR (95% CI) was 1.20 (1.02–1.43) for patients with an ETI > 16 min and <30 min and 1.48 (1.09–2.01) for patients with an ETI of >31 min (*p* for interaction <0.01) compared to ETI < 15 min, used as a reference. **Conclusions**: IV fluid resuscitation improved survival to discharge in OHCA patients, and this benefit was maintained only with the ETI > 16 min.

## 1. Introduction

The acute cessation of blood flow and ventilation in out-of-hospital cardiac arrest (OHCA) is a major public health challenge due to its high morbidity and low survival [1]. The global average incidence in adults is approximately 55 OHCAs per 100,000 person-years, and the survival to discharge rate is lower in Asia (4.5%) than in North America (7.7%) and Europe (11.7%) [2,3].

A key issue in improving OHCA survival is cardiopulmonary resuscitation (CPR), which is aimed at maintaining oxygen delivery to tissues determined by cardiac output and arterial oxygen content [4]. Basic life support (BLS) targets generating cardiac output through chest compression for oxygen delivery and increasing arterial hemoglobin saturation through positive pressure ventilation [5]. In addition to BLS, advanced life support (ALS) aims to enhance oxygen delivery through drugs (epinephrine), defibrillation, and intravenous (IV) fluid resuscitation [6].

IV fluid resuscitation increases the preload and improves cardiac output. In previous studies, IV fluid resuscitation was beneficial in hypovolemic patients with OHCA [7], improved survival to discharge in OHCA children [8], and improved 1-month survival in adult OHCA of non-cardiac etiology [9]. However, the evidence supporting the routine use of isotonic fluid during CPR in OHCA is limited; thus, IV fluid resuscitation is not recommended in CPR guidelines [10,11]. Moreover, animal studies reported that isotonic fluid resuscitation during CPR lowers coronary perfusion pressure (CPP) [12].

Previous studies showed that patients with cardiac arrest invariably develop metabolic acidosis [13,14], and IV fluid resuscitation is helpful in managing acute metabolic acidosis [15]. Therefore, despite a debate about whether routine IV fluid use improves survival in OHCA patients, we hypothesized that the benefit of IV fluid resuscitation increases as metabolic acidosis intensifies while the low flow time increases [16].

This study aimed to determine whether IV fluid resuscitation improves clinical outcomes in OHCA patients and identify how the duration of emergency medical service (EMS)-treated time interval (ETI), including scene time interval and transport time interval, influences the effect of IV fluid resuscitation.

## 2. Materials and Methods

### 2.1. Study Design and Setting

This was a cross-sectional study using a nationwide, population-based registry including all OHCA patients transported by EMS in Korea.

Korea has approximately 50 million people living in 100,210 km^2^ and consists of 220 counties in 17 provinces. The Korean EMS system is a single-tiered, government-based system operated by 17 provincial headquarters of the National Fire Agency (NFA). EMS providers perform basic life support (BLS) and advanced life support (ALS), including mechanical chest compression, advanced airway management (endotracheal intubation and supraglottic airway), IV fluid resuscitation, and IV drug use on-scene and during transport under direct medical control. In the Korean EMS system, 0.9% normal saline (N/S) is administered under the guidance of a medical director during on-scene treatment or transportation unless specific conditions such as hypoglycemia necessitate alternative interventions. Resuscitation termination at the scene is not permitted unless obvious signs of death are confirmed, in addition to permission from the medical control physician.

In Korea, 5 cycles (2 min) of CPR are implemented at the scene with the scoop and run system, followed by transferring the patient to the hospital regardless of return of spontaneous circulation (ROSC). Emergency medical technicians (EMTs) in Korea are classified into level-1 and -2 EMTs (comparable to EMT-intermediated and EMT-basic in the United States, respectively). Ambulance crews usually comprise 3 members (82.4% in 2020), with at least one member who is qualified as a level-1 EMT or nurse who can administer IV fluids and medications under medical control.

Of 226 fire stations, 31 fire stations conducted a smart ALS (SALS) pilot project for transporting patients after prolonged on-scene resuscitation until ROSC or death confirmed with advanced airway management and IV medication, such as epinephrine or amiodarone, under the medical control of emergency physicians [17].

In Korea, there are 402 emergency departments (EDs) categorized into three levels by the government according to the capacity and resources, such as equipment, staffing, and size of ED: level-1 ED (*n* = 38), level-2 ED (*n* = 128), and level-3 ED (*n* = 236). All EDs generally perform advanced cardiovascular life support (ACLS) and post-cardiac arrest (PCA) care according to international standard guidelines [18].

### 2.2. Data Sources

We identified a study population using the Korean nationwide OHCA registry started in 2006 in collaboration with the Korea Centers for Disease Control and Prevention (CDC) and the NFA. The EMS OHCA registry was used to collect patients’ demographics, pre-hospital profiles, and Utstein information. Moreover, a medical record reviewer from the Korean CDC reviewed hospital medical records on etiology, hospital care, and clinical outcomes based on the Utstein guidelines. The OHCA registry quality management committee (QMC) developed the medical record review guidelines. The QMC is composed of emergency medicine physicians, cardiologists, epidemiologists, statistical experts, and medical record review experts. All information, including definitions of variables, inclusion and exclusion criteria, examples, and warnings, is described in the guidelines [19].

### 2.3. Study Population

This study included all EMS-treated OHCA patients aged ≥ 18 years with presumed cardiac etiology between January 2019 and December 2021. Patients for whom resuscitation was not attempted by EMS, who achieved ROSC at the scene, or who were transported by the ambulance that implemented the SALS pilot project were excluded.

### 2.4. Main Outcomes

The primary outcome was survival to hospital discharge and neurological recovery, defined as a Glasgow–Pittsburgh cerebral performance category (CPC) of 1 or 2. The CPC score was determined by the medical record reviewers based on the discharge summary and documentation in the medical records.

### 2.5. Variable and Measurements

The main exposure of this study was whether the IV fluid resuscitation was conducted by an EMS provider during transportation. The secondary exposure was ETI, which is the sum of scene time interval (time from ambulance arrival to departure from the scene) and transport time interval (time from departure to hospital arrival).

Patients’ information on age, sex, comorbidities (diabetes mellitus, hypertension, heart disease, stroke, and kidney disease), urbanization level (metropolitan or urban/rural area), and place of arrest (private or public) were collected. Prehospital-EMS information, including witness status, bystander CPR, initial electrocardiogram (ECG) rhythm (shockable or non-shockable), EMS time variables (response time interval [time from the call to ambulance arrival at the scene], scene time interval, and transport time interval), multi-tier response, the number of EMS providers per transport, mechanical chest compression device, advanced airway management, and epinephrine use was retrieved. Hospital treatment information (reperfusion treatment, extracorporeal CPR, and targeted temperature management) and clinical outcomes were also collected.

### 2.6. Statistical Analysis

We compared patient demographics, characteristics of the cardiac arrest, time intervals and procedures, and study outcomes according to IV fluid resuscitation using the Chi-square test for categorical variables and the Wilcoxon rank-sum test for continuous variables.

Univariable and multivariable logistic regression analyses were performed to estimate the effect sizes of fluid resuscitation on survival outcomes. Crude and adjusted odds ratio (OR and aOR, respectively) with a 95% confidence interval (CI) for the association between IV fluid resuscitation and outcome measures were calculated. The models were adjusted for potential confounders, including patient factors (age, sex, urbanization level, and comorbidities, including diabetes mellitus, hypertension, and heart disease), arrest factors (place of arrest, witness state, bystander CPR, and initial shockable rhythm on ECG identified at the scene), and EMS factors (response time interval, scene time interval, multi-tier response, EMS airway management, and mechanical CPR). We also performed a sensitivity analysis except for those who used IV drugs (epinephrine, lidocaine, and amiodarone). Finally, the interaction between IV fluid resuscitation and ETI (scene time interval plus transport time interval) was analyzed using stratified analysis. All variables in the final model were assessed for multicollinearity, which was not detected in this analysis.

All statistical analyses were performed using SAS version 9.4 (SAS Institute Inc., Cary, NC, USA). All *p*-values were two-tailed, with *p* < 0.05 considered statistically significant.

## 3. Results

**Figure 1:**
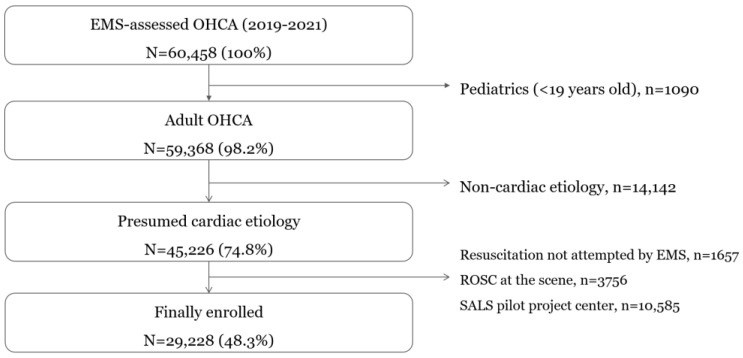
Study population of observational study.

Among 60,458 identified EMS-treated OHCA cases, 29,228 (48.3%) met the inclusion criteria. We excluded patients younger than 18 years (*n* = 1090), those with non-cardiac etiology (*n* = 14,142), in whom resuscitation was not attempted by EMS (*n* = 1657), those with ROSC at the scene (*n* = 3756), and those transported by EMS belonging to a fire station implementing the SALS pilot project (*n* = 10,585) (Figure 1).

### 3.1. Demographic Findings

Table 1 presents patient demographics according to IV fluid resuscitation. Of 29,228 eligible patients, 13,683 (46.8%) OHCA patients were treated with IV fluid resuscitation. The good neurological recovery and survival to discharge rates were 1.8% and 4.3% in the fluid resuscitation group and 2.7% and 5.4% in the non-fluid resuscitation group, respectively (both *p* < 0.01). The fluid resuscitation group showed a longer scene time interval, more multi-tier responses, and more advanced EMS treatment, including mechanical chest compression device use, advanced airway management, and epinephrine use, than the fluid resuscitation group (*p* < 0.01).

### 3.2. Main Results

Table 2 demonstrates the results of the multivariable logistic regression analysis. After adjusting for potential confounders, patients who received IV fluid resuscitation had a significantly higher likelihood of survival to discharge but not significantly different neurological recovery compared to the non-fluid resuscitation group (aOR [95% CI]: 1.15 [1.01–1.32] and 1.10 [0.89–1.36], respectively). Sensitivity analysis, in which patients receiving IV drugs were excluded, showed that IV fluid resuscitation provided significantly higher odds of survival to discharge but not good neurological recovery (aOR [95% CI]: 1.17 [1.02–1.34] and 1.15 [0.93–1.42], respectively).

### 3.3. Stratified Analysis

The stratified analysis showed statistically significant interaction effects between IV fluid resuscitation and ETI (Table 3). The aORs for study outcomes in the fluid resuscitation group differed depending on the ETI compared to the non-fluid resuscitation group. The fluid resuscitation group had an interaction effect for survival to discharge (aOR [95% CI]): 1.20 (1.02–1.43) for patients with an ETI > 16 min and <30 min, 1.48 (1.09–2.01) for patients with an ETI > 31 min (both *p* for interaction <0.01). In sensitivity analysis, the fluid resuscitation group had significantly higher odds of survival to discharge for patients with an ETI > 16 min and <30 min and ETI > 31 min (aOR [95% CI]: 1.24 [1.04–1.48] and 1.74 [1.26–2.41]) (both *p* for interaction <0.01).

## 4. Discussion

Using the Korean National OHCA database, this study discovered that adult OHCA patients with presumed cardiac etiology who received IV fluid resuscitation were more likely to have better survival to discharge compared to patients with no fluid resuscitation. In the stratified analysis, survival to discharge was improved only when the EMS time interval lasted >16 min. This research contributes to understanding the benefit of IV fluid resuscitation for OHCA patients and will help develop strategies to improve survival outcomes in OHCA with long ETI.

International Liaison Committee on Resuscitation (ILCOR), European Resuscitation Council (ERC), and RECOVER ALS guidelines recommend a conservative approach to IV fluid resuscitation during CPR, reserving IV fluid boluses only for patients with known or suspected hypovolemia or distributive shock [7,20,21]. The Australian and New Zealand Committee on Resuscitation recommends that fluids should be infused at a rate of at least 20 mL/kg if hypovolemia is suspected.

The large OHCA registry in Osaka demonstrated that fluid resuscitation increased 1-month survival by 45% in adult OHCA of non-cardiac etiology [8]. In a study of pediatric OHCA, fluid resuscitation was associated with increased survival, whereas resuscitation drugs were not [9]. In a Taiwan study including 27,270 OHCA patients, fluid resuscitation increased survival to discharge; however, judging the effect of fluid alone was difficult because most patients received injection drugs (e.g., inotropes) with the fluid [22]. Many guidelines and studies recommend isotonic fluid resuscitation for hypovolemic OHCA patients; however, resuscitation for euvolemic or all OHCA patients might be detrimental due to decreased tissue blood flow caused by compromised tissue perfusion pressure contrary to the results of our study [7]. IV fluid administration can increase central venous pressure (CVP), which in turn reduces coronary and cerebral perfusion pressures, thereby impairing effective blood flow to vital organs. Additionally, excessive fluid administration may cause right ventricular distension, leading to decreased left ventricular filling and further compromising systemic perfusion. Hyperchloremic acidosis induced by large volumes of 0.9% normal saline may also reduce myocardial contractility and peripheral vascular resistance, exacerbating tissue hypoperfusion. Furthermore, in the metabolic phase of cardiac arrest, where a sepsis-like state with inflammatory responses and capillary leakage prevails, excessive fluid administration may worsen tissue edema and capillary leak syndrome, thereby diminishing tissue oxygenation and perfusion. These mechanisms may explain why fluid resuscitation could be detrimental in some euvolemic patients.

Although 0.9% N/S has been associated with the risk of hyperchloremic acidosis at higher doses, its administration in moderate amounts (1–2 L) is considered safe and effective in most cases, as supported by existing studies. In our study, the average volume of N/S administered was less than 1 L (approximately 866 mL), which minimizes the likelihood of hyperchloremic acidosis while providing the necessary circulatory support to improve metabolic acidosis. Furthermore, assessment of intravascular volume status might be challenging in patients with OHCA.

According to the three-phase time-sensitive model [23], which reflects the time-sensitive progression of resuscitation physiology, the effect of defibrillation and CPR decreases rapidly in about 10 min after cardiac arrest, and the survival rate is poor. During this metabolic phase in the three-phase model, tissue injury from global ischemia and reperfusion can result in circulating metabolic factors causing additional injury beyond the effects of focal ischemia [23]. Additionally, differences in interleukin and tumor necrosis factor levels and immunological changes in resuscitation are similar to those in sepsis [24]. The continuation and progression of such a sepsis-like state as cardiac arrest might explain why fluid resuscitation increased survival outcomes only when the EMS time interval was >16 min in our study.

In our study, the IV fluid group included a higher proportion of patients who received bystander CPR and presented with shockable rhythms, indicating better prognostic potential. These patients were more likely to receive intensive interventions, such as IV fluid therapy, epinephrine administration, and advanced airway management during prehospital care, as well as advanced hospital treatments like TTM, PCI, and ECMO. Despite these efforts, the IV fluid group also had a significantly higher proportion of patients with delayed scene time intervals exceeding 16 min, which is known to negatively impact clinical outcomes. In our analysis, this prolonged scene time interval might have contributed to the worse outcomes observed in unadjusted comparisons. However, after adjusting for scene time interval and other confounders using logistic regression analysis, IV fluid administration was associated with better survival outcomes, particularly for patients with prolonged scene and transport times exceeding 16 min. These findings emphasize the importance of considering time intervals as critical confounding variables when interpreting the effectiveness of IV fluid therapy. This may partly explain why unadjusted comparisons showed worse survival and neurological recovery in the IV fluid group. However, after adjusting for these variables using logistic regression analysis, we found that IV fluid administration was associated with better survival outcomes, particularly in patients with prolonged scene and transport times exceeding 16 min. These findings highlight the importance of accounting for time intervals and other confounding variables when interpreting the outcomes of IV fluid therapy.

Moreover, fluid resuscitation during CPR can theoretically increase tissue driving pressure by elevating aortic diastolic pressure (ADP) and mean arterial pressure (MAP). However, it may simultaneously raise counteracting outflow pressures, such as central venous pressure (CVP) and right atrial diastolic pressure (RADP). This increase in outflow pressure can offset the benefits of higher driving pressure by reducing cerebral perfusion pressure (CePP) and coronary perfusion pressure (CPP), potentially compromising effective blood flow to vital organs [7]. Thus, fluid resuscitation can be administrated only for patients in whom driving pressure (ADP and MAP) is greater than the outflow pressure (CVP and RADP). It is also useful for patients who are hypovolemic, either due to inappropriate vasodilation or intravascular volume loss induced by the sepsis-like state.

Another theory that can explain our finding is the body temperature maintenance effect due to fluid resuscitation. Meta-analysis and randomized controlled trials showed that although intra-arrest cooling did not influence the survival outcomes of OHCA patients, it might influence survival by reducing the time to reach the target temperature [25,26,27]. In our study, the mean amount of fluid resuscitation was 866.3 mL (standard deviation [SD]: 493.8). Although this amount was less than the amount reported by previous intra-arrest cooling studies [28], it might influence the prevention of fever and maintenance of normal body temperature. However, body temperature data, such as tympanic temperature, were not measured in our study, limiting our ability to directly confirm this hypothesis. This suggestion is based on the general effects of fluid resuscitation observed in previous studies rather than data directly obtained in this study.

Since fluid resuscitation might have multicollinearity with other IV drugs, such as epinephrine or amiodarone, sensitivity analysis was performed except for cases where other drugs were administered, showing that the study results did not change.

Although our study result is different from previous studies and guidelines, showing that fluid resuscitation is useful only for patients with proven or expected hypovolemic state, we suggested that isotonic fluid resuscitation might increase survival to discharge when the ETI is >16 min. Our study results can represent a theoretical basis for fluid resuscitation in patients who are expected to have longer scene and transport time intervals, although a well-designed randomized controlled trial is needed. Our study had several limitations. First, our study was not a randomized controlled study. Thus, there might be inconsiderable confounders, and natural development might influence study outcomes, such as by improving the CPR quality of EMS providers. Additionally, all OHCA cases in the Korean EMS system are managed under the medical guidance of a medical director, who determines whether IV fluid administration is necessary and orders EMTs accordingly. However, the medical director’s decision-making lacks standardized, objective criteria, which could introduce bias in the study. This potential bias may partly explain why less than half of the cardiac arrest patients in our study received IV fluids. Furthermore, as a retrospective study, our research inherently has limitations, including potential biases related to data collection and unmeasured confounders. Second, the timing and velocity of fluid resuscitation were not investigated in our registry, and information on the amount of fluid was often missing. Third, etCO_2_ and blood pressure, which are critical indicators of hemodynamic improvement, were not measured during IV fluid resuscitation at the scene or during transport in our study. This limitation stems from the standard EMS practice in our region, where such physiological parameters are not routinely recorded during resuscitation. As a result, our hypothesis regarding the hemodynamic effects of IV fluid resuscitation is based on general evidence from the existing literature rather than direct data from our study. The absence of these measurements limits the ability to confirm the proposed mechanism linking IV fluid resuscitation to improved survival outcomes. Fourth, as this study was retrospective in nature, the conclusions drawn are based on observations rather than explanations of causality. While we attempted to adjust for potential confounders using multivariable analysis, the inherent limitations of a retrospective design make it difficult to establish definitive causal relationships between IV fluid resuscitation and survival outcomes. Prospective or randomized controlled trials are necessary to validate our findings and confirm the causative effects of fluid resuscitation. Fifth, knowing the total no-flow time or low-flow time after the collapse, which was not presented in our study, is necessary for presenting a three-phase model to explain our results. No-flow time is a critical factor influencing survival outcomes in OHCA. While our study utilized prehospital EMS data, which included response time interval, scene time interval, and transport time interval, it did not capture detailed information about no-flow time occurring during or between these phases.

## 5. Conclusions

IV fluid resuscitation for OHCA patients increased survival to discharge rate when the ETI was >16 min. Thus, fluid resuscitation can be considered in OHCA patients expected to have longer ETI.

## Figures and Tables

**Table 1:** Characteristics of out-of-hospital cardiac arrest patients according to the prehospital fluid resuscitation.

	Total	Fluid Resuscitation		
		Yes	No	*p*-Value
	*n* (%)	*n* (%)	*n* (%)	
Total	29,228	13,683	15,545	
Age, year				<0.01
19–65	8384 (28.7)	4199 (30.7)	4185 (26.9)	
65–120	20,844 (71.3)	9484 (69.3)	11,360 (73.1)	
Sex, female	11,017 (37.7)	4730 (34.6)	6287 (40.4)	<0.01
Comorbidity				
Diabetes mellitus	6924 (23.7)	3376 (24.7)	3548 (22.8)	<0.01
Hypertension	10,574 (36.2)	5120 (37.4)	5454 (35.1)	<0.01
Heart disease	5316 (18.2)	2607 (19.1)	2709 (17.4)	<0.01
Stroke	2815 (9.6)	1241 (9.1)	1574 (10.1)	<0.01
Kidney disease	1840 (6.3)	866 (6.3)	974 (6.3)	0.82
Metropolitan area	11,134 (38.1)	7090 (51.8)	4044 (26.0)	<0.01
Place of arrest, public	7115 (24.3)	2962 (21.6)	4153 (26.7)	<0.01
Arrest witnessed	14,189 (48.5)	6459 (47.2)	7730 (49.7)	<0.01
Bystander CPR	16,279 (55.7)	8302 (60.7)	7977 (51.3)	<0.01
Initial shockable rhythm	3536 (12.1)	1942 (14.2)	1594 (10.3)	<0.01
Response time interval, min				<0.01
0–3	1449 (5.0)	655 (4.8)	794 (5.1)	
4–7	15,162 (51.9)	7631 (55.8)	7531 (48.4)	
≥8	12,617 (43.2)	5397 (39.4)	7220 (46.4)	
Median (IQR)	7 (5–10)	7 (5–9)	7 (5–9)	<0.01
Scene time interval, min				<0.01
0–10	8351 (28.6)	2025 (14.8)	6326 (40.7)	
11–15	11,482 (39.3)	5751 (42.0)	5731 (36.9)	
≥16	9395 (32.1)	5907 (43.2)	3488 (22.4)	
Median (IQR)	13 (10–17)	15 (12–18)	15 (12–18)	<0.01
Transport time interval, min				<0.01
0–3	4707 (16.1)	2173 (15.9)	2534 (16.3)	
4–7	11,137 (38.1)	5827 (42.6)	5310 (34.2)	
≥8	13,384 (45.8)	5683 (41.5)	7701 (49.5)	
Median (IQR)	7 (4–11)	7 (4–10)	7 (4–10)	<0.01
Multi-tier response	19,346 (66.2)	11,359 (83.0)	7987 (51.4)	<0.01
EMT number, 3 people	28,267 (96.7)	13,538 (98.9)	14,729 (94.8)	<0.01
EMS management				
Mechanical CPR	6705 (22.9)	4594 (33.6)	2111 (13.6)	<0.01
Advanced airway	22,698 (77.7)	12,205 (89.2)	10,493 (67.5)	<0.01
Epinephrine use	3091 (10.6)	2946 (21.5)	145 (0.9)	<0.01
ED level				<0.01
Level 1	5440 (18.6)	2791 (20.4)	2649 (17.0)	
Level 2	13,361 (45.7)	7091 (51.8)	6270 (40.3)	
Level 3	10,427 (35.7)	3801 (27.8)	6626 (42.6)	
Hospital treatment				
TTM	692 (2.4)	395 (2.9)	297 (1.9)	<0.01
PCI	1173 (4.0)	628 (4.6)	545 (3.5)	<0.01
ECMO	310 (1.1)	196 (1.4)	114 (0.7)	<0.01
Survival outcomes				
Prehospital ROSC	9454 (32.3)	4664 (34.1)	4790 (30.8)	<0.01
Survival to discharge	1433 (4.9)	593 (4.3)	840 (5.4)	<0.01
Good neurological recovery	676 (2.3)	249 (1.8)	427 (2.7)	<0.01

CPR, cardiopulmonary resuscitation; EMT, emergency medical technician; EMS, emergency medical service; ED, emergency department; TTM, targeted temperature management; PCI, percutaneous coronary intervention; ECMO, extracorporeal membrane oxygenation.

**Table 2:** Multivariable logistic regression model for study outcomes.

	Total	Outcome		Model 1			Model 2			Model 3			Model 3 (Fluid Only)		
	*n*	*n*	%	aOR	95% CI		aOR	95% CI		aOR	95% CI		aOR	95% CI	
Good neurological recovery															
IV fluid (−)	15,545	427	2.7	1.00			1.00			1.00			1.00		
IV fluid (+)	13,683	249	1.8	0.61	0.52	0.72	0.64	0.54	0.77	1.10	0.89	1.36	1.15	0.93	1.42
Survival to discharge															
IV fluid (−)	15,545	840	5.4	1.00			1.00			1.00			1.00		
IV fluid (+)	13,683	593	4.3	0.75	0.68	0.80	0.79	0.70	0.89	1.15	1.01	1.32	1.17	1.02	1.34
Prehospital ROSC															
IV fluid (−)	15,545	4790	30.8	1.00			1.00			1.00			1.00		
IV fluid (+)	13,683	4664	34.1	1.14	1.08	1.20	1.15	1.09	1.21	1.25	1.18	1.33	1.26	1.18	1.34

aOR, adjusted odds ratio; CI, confidence interval; IV, intravenous; ROSC, return of spontaneous circulation. Model 1: adjusted for age, gender, cardiac arrest center, urbanization level, and comorbidity (diabetes mellitus, hypertension, and heart disease). Model 2: adjusted for variables in Model 1 plus place of arrest, witness status, bystander CPR, and initial shockable rhythm. Model 3: adjusted for variables in Model 2 plus response time interval, scene time interval, multi-tier response, EMS airway management, and mechanical CPR.

**Table 3:** Stratified analysis between fluid resuscitation and EMS time interval (STI and TTI).

		IV Fluid (−)	IV Fluid (+)			
		aOR	aOR	95% CI		*p* Value
Whole population						
Good neurological recovery						
EMS time interval						0.21
	≤15 min	ref.	0.81	0.54	1.20	
	16–30 min	ref.	1.21	0.93	1.57	
	≥31 min	ref.	1.19	0.79	1.79	
Survival to discharge						
EMS time interval						<0.01
	≤15 min	ref.	0.87	0.67	1.14	
	16–30 min	ref.	1.20	1.02	1.43	
	≥31 min	ref.	1.48	1.09	2.01	
Subgroup (fluid only) population						
Good neurological recovery						
EMS time interval						0.15
	≤15 min	ref.	0.91	0.61	1.36	
	16–30 min	ref.	1.19	0.90	1.58	
	≥31 min	ref.	1.61	1.05	2.48	
Survival to discharge						
EMS time interval						<0.01
	≤15 min	ref.	0.90	0.69	1.18	
	16–30 min	ref.	1.24	1.04	1.48	
	≥31 min	ref.	1.74	1.26	2.41	

STI, scene time interval; TTI, transport time interval; IV, intravenous; EMS, emergency medical service.

## Data Availability

The data of this study were obtained from the Korean Centers for Disease Control and Prevention, but restrictions apply to the availability of these data, so they are not publicly available.
